# Comparison of the Safety of Proton Pump Inhibitors and Histamine Type 2-Receptor Antagonists in the Prevention of Gastrointestinal Complications

**DOI:** 10.7759/cureus.103329

**Published:** 2026-02-10

**Authors:** Angelika Samborska, Marta Karczewska, Karolina Lichwala, Sara Szukalska, Kamil Wróblewski, Lukasz Siwek, Barbara Balajewicz

**Affiliations:** 1 General Medicine, Medical University of Silesia, Katowice, POL; 2 Orthopaedics and Traumatology, Radomski Szpital Specjalistyczny im. dr. Tytusa Chałubińskiego w Radomiu, Radom, POL; 3 General Medicine, University Clinical Hospital, Poznan University of Medical Sciences, Poznan, POL; 4 Medicine, Szpital Miejski w Zabrzu, Zabrze, POL

**Keywords:** h2 receptor antagonists, prevention of gastrointestinal complications, proton pump inhibitors, safety of pharmacotherapy, upper gastrointestinal bleeding

## Abstract

This study aims to compare the efficacy and safety of proton pump inhibitors (PPIs) and histamine type 2-receptor antagonists (H2RAs) in preventing gastrointestinal (GI) complications, particularly bleeding, in high-risk patient populations, including ICU patients and individuals taking aspirin, dual antiplatelet therapy (DAPT), or nonsteroidal anti-inflammatory drugs (NSAIDs). A literature review was conducted following Preferred Reporting Items for Systematic Reviews and Meta-Analyses (PRISMA) and Cochrane-aligned principles. Meta-analyses, systematic reviews, randomized controlled trials, and large cohort studies published between 1997 and 2025 were included. Searches were performed in PubMed, Cochrane Library, MEDLINE, Embase, and SpringerLink to identify studies comparing PPIs and H2RAs with respect to prophylactic effectiveness and adverse outcomes, including infections, pneumonia, and mortality. Data were synthesized narratively with emphasis on studies of higher methodological quality.

Overall, the reviewed evidence generally suggested that PPIs reduce clinically significant bleeding more effectively than H2RAs in ICU settings and in patients receiving aspirin or DAPT. Among NSAID users, PPIs were also more effective for ulcer healing and prevention. In contrast, several analyses indicated higher rates of certain adverse events among PPI recipients, particularly infections and possibly pneumonia, while H2RAs appeared to have a more favorable safety profile for these outcomes. Mortality findings were inconsistent across studies, with some large trials reporting no meaningful differences between drug classes. In conclusion, PPIs may offer greater protection against bleeding and ulceration than H2RAs but can be associated with a higher risk of selected adverse events. Prophylactic therapy should be individualized by balancing expected benefits with patient-specific risks, including infection risk and concurrent antiplatelet therapy.

## Introduction and background

Upper gastrointestinal (GI) complications, including stress-related mucosal disease and bleeding, represent a significant clinical concern in hospitalized patients, particularly those treated in ICUs [1,2]. According to available evidence, gastric prophylaxis is most commonly applied in critically ill patients with major risk factors, particularly those requiring prolonged mechanical ventilation, and in patients receiving high-dose corticosteroids with established risk factors for stress-related mucosal injury [1,2]. In this review, GI complications of interest include stress-related mucosal disease, erosive gastritis, peptic ulcer disease, upper GI bleeding (including overt and clinically significant bleeding), nonsteroidal anti-inflammatory drug (NSAID)-associated ulcers, and aspirin- or antiplatelet-related gastroduodenal injury [1,2].

Proton pump inhibitors (PPIs) and histamine type 2-receptor antagonists (H2RAs) are the primary agents used for prophylaxis, differing in mechanism of action and potential clinical performance [3,4]. Proton pump inhibitors suppress gastric acid secretion by irreversibly inhibiting the H⁺/K⁺-ATPase in gastric parietal cells, whereas histamine-2 receptor antagonists reduce acid production through competitive blockade of H2 receptors, resulting in a less potent and shorter-lasting acid-suppressive effect [1,2]. Although PPIs are considered more potent acid-suppressive agents, available research provides mixed evidence regarding their clinical superiority over H2RAs in preventing bleeding and ulceration [5,6]. The safety profiles of both drug classes also differ, with some studies indicating increased risks of infectious complications associated with PPIs [7,8], particularly *Clostridioides difficile* infection and pneumonia, as well as other non-infectious adverse effects, including electrolyte disturbances (e.g., hypomagnesemia), vitamin B12 deficiency, renal adverse effects such as acute interstitial nephritis and an increased risk of chronic kidney disease, and clinically relevant drug-drug interactions [9].

Upper GI bleeding remains a frequent and clinically relevant complication in hospitalized and critically ill patients. The reported incidence of stress-related GI bleeding in ICU populations ranges from approximately 2% to 6%, with clinically significant bleeding occurring in 1% to 4% of patients, depending on illness severity and prophylactic strategies employed [1,2,5]. Importantly, upper GI bleeding is associated with substantial morbidity and mortality, with reported mortality rates ranging from approximately 5% to 10% in the general hospitalized population and exceeding 20% in critically ill patients, particularly those with multiple comorbidities or hemodynamic instability [1,2,5].

In patients receiving antiplatelet therapy or NSAIDs, the risk of upper GI bleeding is further increased, especially among older individuals and those with a history of peptic ulcer disease [1,2,5]. Infectious complications associated with acid-suppressive therapy have also been widely reported. A *C. difficile* infection occurs in approximately 1% to 5% of hospitalized patients, with higher rates observed in critically ill populations and in those exposed to prolonged PPI therapy [7,8]. Several observational studies have suggested an increased risk of pneumonia among hospitalized and ICU patients receiving acid-suppressive therapy, although the strength and clinical relevance of this association remain variable across studies [3,4]. Current clinical guidelines support the use of either PPIs or H2RAs, recommending individualized therapy based on patient-specific risk factors [9].

## Review

Methods

This study was conducted as a narrative review incorporating elements of a systematic review and following established methodological principles for evaluating clinical and observational evidence. The objective was to compare the efficacy and safety of PPIs and H2RAs in preventing upper GI complications across multiple patient groups, including critically ill patients and those taking low-dose aspirin (LDA), dual antiplatelet therapy (DAPT), and NSAIDs. A structured literature search was conducted in PubMed, MEDLINE, Embase, Cochrane Library, SpringerLink, ScienceDirect, and full-text repositories (PLOS, BMC, PMC). Studies published between 1997 and 2025, written in English, and available with full text were included. Search strategies used combinations of the following terms: “proton pump inhibitors,” “H2 receptor antagonists,” “stress ulcer prophylaxis,” “upper gastrointestinal bleeding,” “low-dose aspirin,” “dual antiplatelet therapy,” “NSAID-associated ulcers,” “Clostridioides difficile,” and “pneumonia risk.” Included studies comprised meta-analyses and systematic reviews directly comparing PPIs and H2RAs [1,2,5,10-14], randomized controlled trials (RCTs) comparing PPIs and H2RAs in ICU, LDA, DAPT, and NSAID populations [6,15-20], observational and cohort studies reporting risks ofinfection or bleeding [7,8,21,22], and clinical guidelines, including Society of Critical Care Medicine (SCCM) and American Society of Health-System Pharmacists (ASHP) recommendations [9].

**Figure 1:**
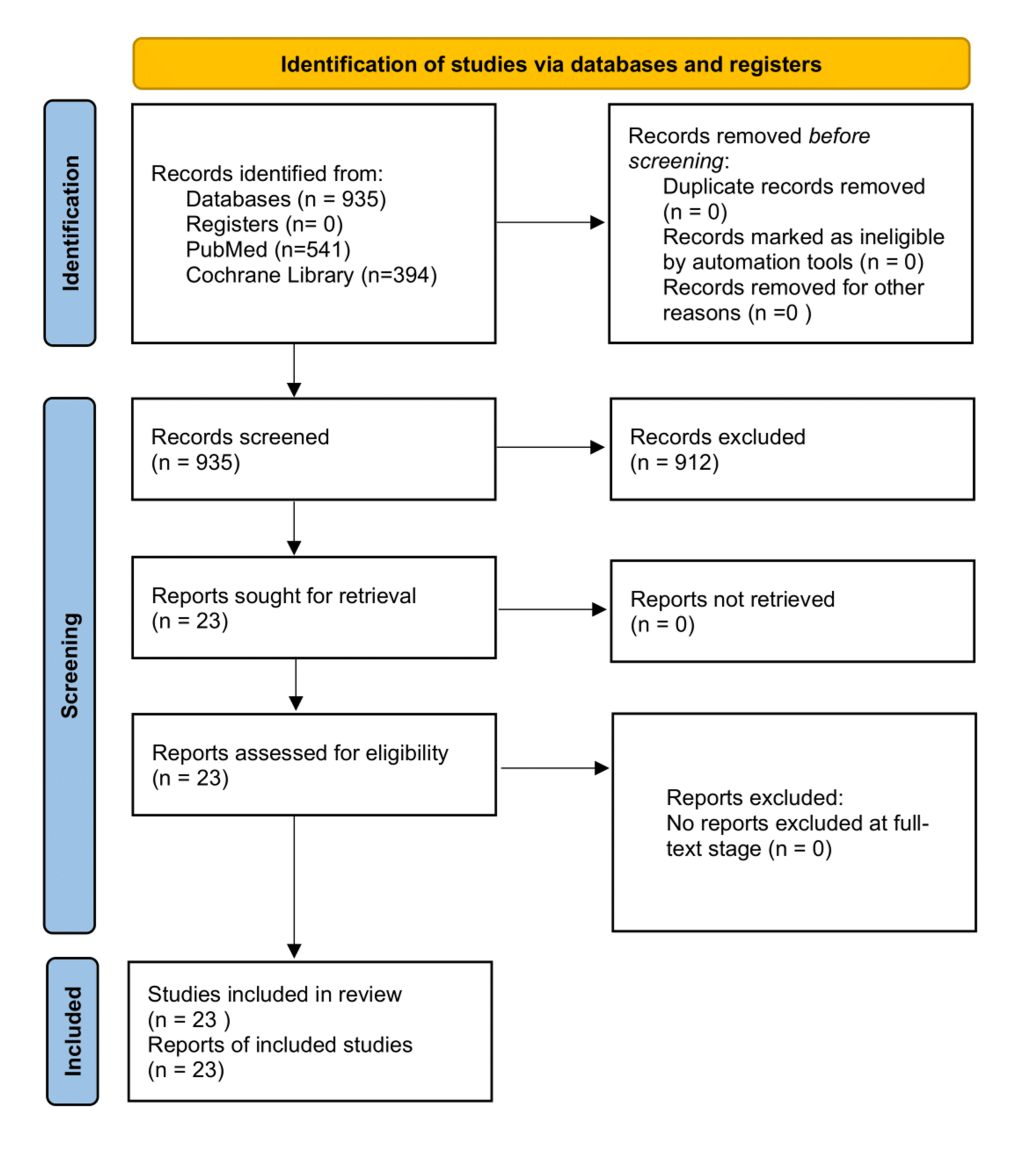
PRISMA 2020 flow diagram Records were identified through database searching, screened for eligibility, and assessed according to predefined inclusion and exclusion criteria. The diagram was adapted from the PRISMA 2020 statement (Creative Commons Attribution 4.0 International License) [23]. PRISMA: Preferred Reporting Items for Systematic Reviews and Meta-Analyses

Exclusion criteria comprised pediatric studies, commentaries, editorials, incomplete reports, noncomparative studies, and studies with insufficient methodological quality. Study selection, screening, and reporting were conducted in accordance with the Preferred Reporting Items for Systematic Reviews and Meta-Analyses (PRISMA) 2020 guidelines [23]. Across the included studies, acid-suppressive therapies were administered using a range of dosing regimens reflecting both prophylactic and therapeutic clinical practice. Proton pump inhibitors were used in standard prophylactic doses (e.g., omeprazole 20 mg daily or equivalent) as well as in higher therapeutic doses (e.g., omeprazole 40-80 mg daily or equivalent), particularly in critically ill patients, those with active or recent upper GI bleeding, and patients receiving NSAIDs or antiplatelet therapy [1,2,5,6,11,19,20]. Similarly, H2RAs were administered in standard prophylactic doses (e.g., ranitidine 150 mg daily or famotidine 20 mg daily) and, in some studies, in higher therapeutic doses in patients at increased risk of GI bleeding [6,15-17]. Variability in dosing strategies was considered a potential contributor to differences in efficacy and safety outcomes between drug classes and was taken into account during qualitative synthesis of the evidence. Figure 1 features the PRISMA flow diagram illustrating the study selection process for the narrative review.

Study quality was evaluated using the assessment of multiple systematic reviews (AMSTAR) for meta-analyses [24], the Cochrane risk of bias tool [25] for RCTs, and methodological criteria addressing confounding control and sample size for observational studies. Due to heterogeneity in populations, outcomes, and methodologies, a narrative synthesis was performed across thematic categories: efficacy of PPIs versus H2RAs in stress ulcer prophylaxis, effectiveness in LDA, DAPT, and NSAID populations, safety outcomes (*C. difficile* infection, pneumonia, and mortality), and comparison of randomized and observational evidence. This study did not employ any proprietary tools, scales, scoring systems, or questionnaires. All data were derived from previously published studies, and no licensed instruments were used.

Results 

A comprehensive body of evidence, including RCTs, meta-analyses, systematic reviews, and observational cohort studies, was analyzed to compare the efficacy and safety of PPIs and H2RAs across multiple clinical contexts. Due to heterogeneity in study designs, patient populations, definitions of drug exposure, and outcome measures, results were synthesized narratively. Findings were organized by clinical context, including GI bleeding, ulcer prevention, NSAID and antiplatelet exposure, infection risk, and adverse outcomes. As this manuscript is a narrative review, the included literature was highly heterogeneous with respect to study design, patient populations, dosing regimens, and reported outcomes. Consequently, not all identified studies were suitable for a structured tabular presentation of characteristics in Table 1. The remaining evidence is therefore synthesized narratively to ensure clarity and coherence of the review.

**Table 1:** Overview of characteristics from included studies This table was intentionally limited to six studies to provide a concise and representative overview of the most methodologically robust and clinically relevant publications that directly compared PPIs and H2RAs. GI: Gastrointestinal, PPI: Proton pump inhibitor, H2RA: Histamine type 2-receptor antagonist

Author (year)	Study design	Study population	Sample size (or no. of studies)	Intervention/ comparator(s)	Clinical setting	Main outcomes
Alhazzani et al. (2018) [1]	Network meta-analysis	Critically ill adults	>13,000	PPI vs H2RA vs placebo	ICU	Clinically significant GI bleeding, mortality
Toews et al. (2018) [2]	Systematic review & meta-analysis	Hospitalized patients	>17,000	PPI vs H2RA	ICU/non-ICU	Upper GI bleeding, adverse events
Suzuki et al. (2020) [3]	Retrospective cohort	Septic shock patients	1,032	PPI vs H2RA	ICU	GI bleeding, mortality
Song et al. (2021) [4]	Systematic review	Critically ill patients	12 studies	PPI vs H2RA	ICU	Stress-related GI bleeding
Wijarnpreecha et al. (2017) [26]	Meta-analysis	Adult population	>500,000	PPI/H2RA exposure	Mixed	Chronic kidney disease
Zeng et al. (2024) [27]	Population-based cohort	Adult population	>1,000,000	PPI vs non-PPI	Community/hospital	Pneumonia, influenza, COVID-19

Critically ill patients receiving stress ulcer prophylaxis

Multiple systematic reviews and meta-analyses consistently demonstrated that PPIs are more effective than H2RAs in preventing clinically significant upper GI bleeding among critically ill patients. Across the included RCTs and observational studies, PPIs were administered using standard prophylactic and therapeutic dosing regimens. In critically ill patients, commonly used PPI doses included omeprazole or pantoprazole 20 mg to 40 mg once daily, with some ICU studies employing higher doses or twice-daily regimens in patients at particularly high risk of bleeding. The H2RAs were most frequently administered as ranitidine 150 mg twice daily or famotidine 20 mg to 40 mg daily, either orally or intravenously in intensive care settings. In studies involving aspirin, DAPT, or NSAID exposure, standard once-daily PPI dosing was most commonly used, whereas higher therapeutic doses were used in ulcer-healing trials.

Due to heterogeneity in dosing strategies across studies, direct dose-response comparisons were not uniformly feasible [1,2,5,6,15,19]. Meta-analyses by Alhazzani et al. and Toews et al. reported that patients receiving PPIs had significantly lower rates of overt and clinically important bleeding than those receiving H2RAs [1,2]. Absolute risk reductions ranged from 2% to 5% in pooled analyses, with relative risk estimates favoring PPIs (risk ratio (RR) ~0.60-0.75). Randomized and observational studies conducted in ICUs supported these findings, consistently showing fewer episodes of clinically significant bleeding with PPI therapy compared with H2RAs [3,4]. An additional meta-analysis by Deliwala et al. also reported lower rates of clinically significant bleeding among patients receiving PPIs than among those treated with H2RAs [5]. Despite these benefits in bleeding reduction, mortality outcomes were less consistent.

The PEPTIC (Proton Pump Inhibitors vs. Histamine-2 Receptor Blockers for Ulcer Prophylaxis Treatment in the Intensive Care Unit) RCT, which enrolled over 26,000 ICU patients across multiple centers, did not demonstrate a statistically significant difference in in-hospital mortality between patients receiving PPIs and those receiving H2RAs [6]. Other analyses reported variable mortality outcomes, while some observational studies suggested increased mortality among PPI users; pooled analyses and large trials failed to confirm a consistent survival disadvantage for either drug class [6,10].

LDA users

Among patients taking LDA, PPIs generally demonstrated superior efficacy over H2RAs in preventing aspirin-related GI complications. A meta-analysis by Mo et al. showed significantly lower rates of erosions, ulcers, and GI bleeding in PPI-treated patients compared to those receiving H2RAs [11]. However, heterogeneity was observed in patients with prior ulcer history. Randomized controlled trials in high-risk aspirin users demonstrated improved mucosal healing with PPI therapy compared with H2RAs [15]. Chan et al. reported that PPIs and H2RAs were similarly effective in preventing recurrent bleeding, suggesting that baseline GI risk factors may influence comparative effectiveness [16]. In addition, PPI treatment was associated with a reduced recurrence of erosions or ulcers during follow-up in aspirin-treated patients [17]. Pooled analyses indicated that the number needed to treat (NNT) to prevent one ulcer-related event was lower for PPIs (NNT ~12-15) than for H2RAs (NNT ~25-30), particularly in older patients and those with concomitant comorbidities such as diabetes or renal insufficiency.

DAPT recipients

In patients receiving DAPT therapy, the literature has shown that PPIs provide stronger protection against upper GI complications than H2RAs. Systematic reviews and randomized comparisons reported lower rates of GI bleeding and mucosal injury among PPI-treated patients [12,18]. For instance, pooled event rates for upper GI bleeding ranged from 1.5% among PPI users to 4.2% among H2RA users, with relative risk reductions of around 60%. No consistent evidence indicated an increased incidence of major adverse cardiovascular events associated with PPI therapy. Nevertheless, some studies reported higher rates of high on-treatment platelet reactivity among PPI users, potentially influencing antiplatelet efficacy in specific contexts [12]. Observational and randomized data suggested that H2RAs may represent a viable alternative for bleeding prophylaxis in patients at higher cardiovascular risk or with a need to minimize drug-drug interactions.

NSAID-exposed populations

Among NSAID-exposed patients, PPIs consistently outperformed H2RAs in both ulcer prevention and healing of established lesions. Multiple RCTs demonstrated higher ulcer healing rates with PPI therapy (e.g., 85% to 92% at eight weeks) compared to H2RA therapy (60% to 75%) [19,20]. Direct head-to-head comparisons, such as omeprazole versus ranitidine trials, consistently favored PPI therapy in terms of mucosal protection, ulcer recurrence reduction, and symptom relief [19]. The effect was more pronounced in high-risk subgroups, including older patients, those with prior GI bleeding, and patients concurrently taking corticosteroids. 

Safety outcomes

Safety data varied across study designs and outcome measures. Observational studies indicated a potential association between PPI use and increased risk of *C. difficile* infection, with some reporting dose-dependent effects [7,8,14,21,22]. Meta-analyses restricted to randomized trials, however, did not demonstrate statistically significant associations between PPI use and *C. difficile* infection, suggesting potential confounding in observational data [7,13,22].

The risk of pneumonia was inconsistently reported; some observational studies found increased incidence among PPI users, while others found no significant difference compared with H2RAs [22]. However, a large population-based cohort study published in 2024 demonstrated that PPI use was associated with an increased risk of pneumonia and influenza [24]. Mortality outcomes also varied; certain observational analyses suggested higher mortality in PPI-treated ICU patients, whereas large RCTs and pooled analyses did not consistently confirm these findings [1,5,6]. Subgroup analyses examining therapy duration revealed that longer-term PPI use (>6 months) did not significantly increase mortality in most RCTs, although some observational studies suggested a higher incidence of adverse events such as renal impairment and electrolyte disturbances, most commonly hypomagnesemia and, less frequently, hypocalcemia and hypokalemia, in prolonged therapy cohorts [21,25].

Mechanistic insights and pharmacodynamic considerations

Proton pump inhibitors irreversibly inhibit the H⁺/K⁺ ATPase proton pump, resulting in profound and sustained gastric acid suppression, whereas H2RAs act as competitive antagonists of histamine-mediated acid secretion. This mechanistic distinction likely explains the superior efficacy of PPIs in high-risk populations, particularly those requiring continuous acid suppression, such as ICU patients, NSAID users, and patients on antiplatelet therapy [1,5,6,9,12].

Dose-response analyses in both RCTs and observational studies suggested that a higher PPI dose range may confer incremental benefits in ulcer healing and bleeding prevention, though higher cumulative exposure was sometimes associated with increased risk of C. difficile infection and metabolic disturbances [14,19,20]. Across studies, H2RAs maintained a favorable safety profile, with fewer reported associations with infection or long-term adverse outcomes, but were generally less effective for high-risk GI protection [1,5,6,10].

Special populations

Several studies examined special patient populations. In elderly patients, PPIs consistently demonstrated superior protection against GI bleeding compared to H2RAs, with absolute risk reductions amplified in those with comorbidities or polypharmacy [11,16]. In patients with chronic kidney disease, available observational data suggest that PPI therapy requires careful monitoring because of potential renal adverse effects; notably, a 2017 meta-analysis demonstrated a statistically significant 1.3-fold increased risk of chronic kidney disease among PPI users compared with those receiving H2RAs [25]. Despite this, the comparative efficacy of PPIs for ulcer prevention remained higher than that of H2RAs. Postoperative ICU patients similarly showed a reduced incidence of stress-related mucosal bleeding with PPI therapy [1,3,6,9].

Across all studies, the comparative efficacy of PPIs and H2RAs showed some heterogeneity, influenced by baseline risk factors, prior GI history, concomitant medications, and therapy duration. Nevertheless, PPIs consistently provided superior outcomes in high-risk populations, particularly in terms of clinically significant GI bleeding, ulcer prevention, and mucosal healing. 

## Conclusions

Existing meta-analyses, RCTs, and large observational studies demonstrate that PPIs are generally more effective than H2RAs in preventing stress-related mucosal disease, erosions, and upper GI bleeding across a broad range of clinical populations, including critically ill patients, individuals on LDA or antiplatelet therapy, and chronic NSAID users. At the same time, the overall risk of severe adverse events, such as mortality, appears comparable between the two drug classes. However, PPIs are consistently associated with a higher risk of infections, particularly *C. difficile*, and, in some analyses, pneumonia. These risks may depend on treatment duration and dosage and are less evident in randomized trials than in observational studies, suggesting the influence of residual confounding. The H2RAs may therefore represent a safer alternative in patients at elevated risk of infectious complications or when the benefits of PPIs do not clearly outweigh their potential harms. Overall, PPIs remain the most effective option for preventing GI complications, particularly in high-risk patients. Nevertheless, therapeutic decisions should be individualized, balancing efficacy with safety considerations. Further high-quality comparative research is warranted, particularly regarding long-term safety and optimal prophylactic strategies in vulnerable populations.
